# Adolescent birth and child undernutrition: an analysis of demographic and health surveys in Bangladesh, 1996–2017

**DOI:** 10.1111/nyas.14608

**Published:** 2021-05-14

**Authors:** Phuong Hong Nguyen, Samuel Scott, Long Quynh Khuong, Priyanjana Pramanik, Akhter Ahmed, Sabina Faiz Rashid, Kaosar Afsana, Purnima Menon

**Affiliations:** ^1^ International Food Policy Research Institute Washington DC; ^2^ Hanoi University of Public Health Hanoi Vietnam; ^3^ James P Grant School of Public Health BRAC University Dhaka Bangladesh

**Keywords:** adolescent birth, early marriage, stunting, underweight, Bangladesh

## Abstract

Adolescent birth is a major global concern owing to its adverse effects on maternal and child health. We assessed trends in adolescent birth and examined its associations with child undernutrition in Bangladesh using data from seven rounds of Demographic and Health Surveys (1996–2017, *n* = 12,006 primiparous women with living children <5 years old). Adolescent birth (10–19 years old) declined slowly, from 84% in 1996 to 71% in 2017. Compared with adult mothers (≥20 years old), young adolescent mothers (10–15 years old) were more likely to be underweight (+11 pp), have lower education (−24 pp), have less decision‐making power (−10 pp), live in poorer households (−0.9 SD) with poorer sanitation (−15 pp), and have poorer feeding practices (10 pp), and were less likely to access health and nutrition services (−3 to −24 pp). In multivariable regressions controlled for known determinants of child undernutrition, children born to adolescents had lower height‐for‐age Z‐scores (−0.29 SD for young and −0.10 SD for old adolescents (16–19 years old)), weight‐for‐age Z‐score (−0.18 and −0.06 SD, respectively) as well as higher stunting (5.9 pp) and underweight (6.0 pp) than those born to adults. In conclusion, birth during adolescence, a common occurrence in Bangladesh, is associated with child undernutrition. Policies and programs to address poverty and improve women's education can help delay marriage, reduce early childbearing, and improve child growth.

## Graphical abstract

Adolescent birth is a major global concern owing to its adverse effects on maternal and child health. We assessed trends in adolescent birth and examined its associations with child undernutrition in Bangladesh using data from seven rounds of Demographic and Health Surveys. Our analysis demonstrates that birth during adolescence, a common occurrence in Bangladesh, is associated with child undernutrition.

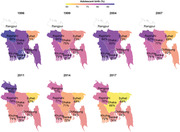

## Introduction

A growing number of countries have recognized the importance of addressing adolescents’ needs in the “leave no one behind” Sustainable Development Goals (SDGs) era.^1^ Central to the list of adolescent issues that the SDGs aim to address are the problems of teenage marriage, pregnancy, and motherhood, which are highly prevalent in South Asian and African countries.^2^ Bangladesh ranks second globally, behind India, in terms of the burden of child marriage and fourth in terms of the prevalence of child marriage; in 2017–2018, 59% of Bangladeshi women 20–24 years old (4.45 million individuals) were married before they were 18 years old.^3^, ^4^ Cultural pressures underpin a short marriage–pregnancy interval in Bangladesh.^5^ In 2018, 69% of Bangladeshi women 20–24 years old had their first birth during adolescence (<20 years old).^6^

It is well established that adolescence is a critical phase of social, physical, and cognitive development.^5^, ^7^ Pregnancy and birth during this phase has far‐ranging consequences for the adolescent herself and for her child, including decreased maternal nutritional status,^8^, ^9^, ^10^ poor access to nutrition and health services,^11^, ^12^ increased maternal morbidity and mortality,^13^, ^14^ and poor pregnancy and child outcomes.^15^, ^16^ Among the long‐term consequences of children born to adolescent mothers is reduced physical growth, including stunting and underweight,^9^ which have been associated with poorer outcomes in childhood and beyond.^17^ Thus, adolescent birth is a key contributor to the intergenerational cycle of growth failure,^18^ and interventions targeting young women entering their childbearing years are an opportunity to reduce child undernutrition.

We recently reported that giving birth during adolescence is associated with poorer outcomes for Indian women—in terms of women's nutrition, access to health services, child feeding practices, living conditions, and education—and with undernutrition in their children.^9^ Yet, India and Bangladesh have very different contexts, with different cultures, health systems, gender norms, dietary habits, and so on. Early marriage is more common in Bangladesh (59%) compared with India (41%), and younger adolescents may face a different set of challenges during pregnancy and postpartum than older adolescents. Thus, adolescent birth may affect women and children differently in Bangladesh compared with India; if such was the case, a different package of interventions would be needed in Bangladesh than in India.

The objectives of the current analysis were (1) to assess trends in adolescent birth and child undernutrition in Bangladesh in the last two decades; (2) examine how adolescent birth explains variability in known determinants of child undernutrition, including maternal nutritional status, education, living conditions, access to health services, and complementary feeding; and (3) examine associations between maternal age at first birth and child undernutrition outcomes accounting for known determinants of child undernutrition.

## Materials and methods

### Data sources

This study used data from seven rounds of data from the Bangladesh Demographic and Health Surveys (BDHS 1996–2017).^4^, ^19^, ^20^, ^21^, ^22^, ^23^, ^24^ The BDHS surveys all used the same two‐stage stratified sampling design covering all administrative divisions. Each division is divided into zilas, and each zila into upazilas. The upazila is further subdivided into wards and mohallas in urban areas as well as union and mouzas in rural areas; these units served as the primary sampling units (enumeration areas, EAs). In the first stage, EAs were selected with probabilities proportional to size. A complete household listing operation was then carried out in all selected EAs to provide a sampling frame for the second‐stage selection of households. In the second stage of sampling, a systematic random sample of 30 households on average per EA was selected. Collected every 3–5 years, BDHS data are representative at both national and division levels and provide extensive data on maternal and child health and nutrition. Datasets are openly published and can be downloaded from the DHS website (https://www.dhsprogram.com/) after obtaining permission from the DHS program. Full details of the survey protocol, sampling procedures, and questionnaires are provided in the BDHS reports.^4^, ^19^, ^20^, ^21^, ^22^, ^23^, ^24^

The sample selection process for this study is presented in Table S1 (online only). In total, 93,334 households were surveyed across all rounds, among them 31,845 mothers with living children born in the last 5 years and having anthropometric data. For this paper, to avoid biases associated with parity and birth spacing, we only included primiparous women who had given birth in the previous 5 years (*n* = 12,006).^11^

### Variables

The outcome variables of interest in this study were child anthropometric measures. Enumerators were trained to measure height/length and weight according to the internationally recommended standard protocol.^25^ Weight was measured using a SECA digital scale. Recumbent length was measured for children <24 months old and standing height was measured for children ≥24 months old using an infant/child Shorr Board. Children's weight and length/height measurements were then used to derive Z‐scores by comparing each child's anthropometric measurements with the WHO age‐ and gender‐appropriate child growth standards.^26^ Three indicators were calculated: length/height‐for‐age Z‐score (HAZ), weight‐for‐age Z‐score (WAZ), and weight‐for‐length/height Z‐score (WHZ). Stunting was defined as HAZ < −2; underweight as WAZ < −2, and wasting as WHZ < −2.^26^

Women's age at first birth was calculated as the difference between the birth date of the first‐born living child and the birth date of the woman. Adolescent birth was defined as women giving first birth between the age of 10–19 years old.^27^ Given evidence of higher risk for giving birth in younger adolescents, we classified women's age at first birth into three categories: 10–15 years old (young adolescent), 16–19 years old (old adolescent), and ≥20 years old (adult).

We used an existing conceptual framework^9^ to select a set of controls, known determinants of child undernutrition, when examining the association between maternal age at first birth and child undernutrition. These determinants are maternal nutrition, women's status, living conditions, access to health services, and infant and young child feeding (IYCF) practices. To measure maternal nutritional status, we used maternal weight and height to calculate body mass index (BMI), with low BMI defined as BMI < 18.5 kg/m^2^. Women's status was measured using completed years of education and bargaining power (work for pay in the last 12 months and ability to make decisions on spending her own earnings, health care, household purchases, and visiting family or relatives). Living condition was measured by improved sanitation facility and household socioeconomic status (SES, which was constructed using principal component analysis, using multiple variables including housing structure and ownership of assets and livestock^28^). Access to nutrition and health services was measured across the continuum of care, including during pregnancy (at least four antenatal care visits), at birth (institutional birth and skilled birth attendant), during postpartum (postnatal care), and during childhood (full immunization, child vitamin A and IFA supplementation, and child deworming). IYCF practices were assessed using standard WHO indicators,^29^ including: early initiation of breastfeeding (the proportion of infants breastfed within 1 h of birth); exclusive breastfeeding (the proportion of infants 0–5.9 months of age fed only breast milk); adequate diet (children who consumed at the minimum meal frequency and at least four of seven food groups in the previous 24 h); and consumption of iron‐rich food.

### Data analysis

Descriptive analyses were conducted for each survey round and regression analyses were done using pooled data from all survey rounds. To examine trends in age at first birth and child nutritional status between 1996 and 2017, we calculated the weighted prevalence of adolescent birth and average values of child anthropometry in each survey round, adjusted for survey‐specific sampling weights using the *svyset* Stata command. We then tested for overall trends over time using a regression model. We used maps to illustrate subnational variability in the prevalence of adolescent birth and child stunting for different survey rounds.

To examine the association between adolescent birth and known determinants of child undernutrition, we applied multivariable regression models with age group at birth as the predictor and the known determinants as outcomes. Models predicting determinants at maternal levels adjusted for maternal religion, survey year, and division fixed effects. Models predicting determinants at child level adjusted for child age, gender, birth order, maternal religion, survey year, and division fixed effects. All models controlled for the cluster sampling design and sampling weights used in the survey. While most data were available for all seven BDHS rounds, some variables were only available for the last five rounds (child vitamin A supplementation), four rounds (adequate diet, postnatal care, and women decision making), or three rounds (child IFA supplementation, child deworming, and iron‐rich foods). In case data were not available for all rounds, the sample size was smaller for the models containing those variables.

To determine the extent to which adolescent birth is associated with child nutritional status, we conducted multivariable regression analyses using full‐information maximum likelihood for estimation while accounting for missing data among the controlled variables under the assumption of missing at random and without having to do imputation.^30^ We compared child anthropometry outcomes between mothers who first gave birth as young adolescents (10–15 years old) or as old adolescents (16–19 years old) with those who first gave birth as adults (≥20 years old), the reference group. The models adjusted for child age, gender, mother's religion, known determinants of undernutrition (including maternal nutritional status, education, living conditions, access to health services, and complementary feeding), and cluster sampling design and sampling weights used in the survey. We also controlled for division fixed effects to account for heterogeneity in factors at the division level such as specific programs or policies that may affect maternal and child health.

All analyses were conducted using Stata® 16 (StataCorp LLC, College Station, TX). Statistical significance was considered at three levels: *P* < 0.05, *P* < 0.01, and *P* < 0.001.

## Results

### Trends in adolescent birth in Bangladesh between 1996 and 2014

**Figure 1:**
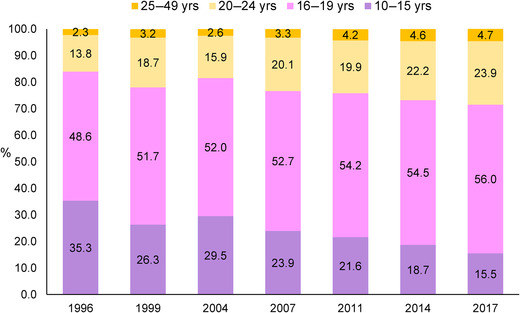
Prevalence of first births by age group among Bangladeshi women from 1996 to 2017. Data were from seven rounds of Bangladesh Demographic and Health Surveys in 1996, 1999, 2004, 2007, 2011, 2014, and 2017.

**Figure 2:**
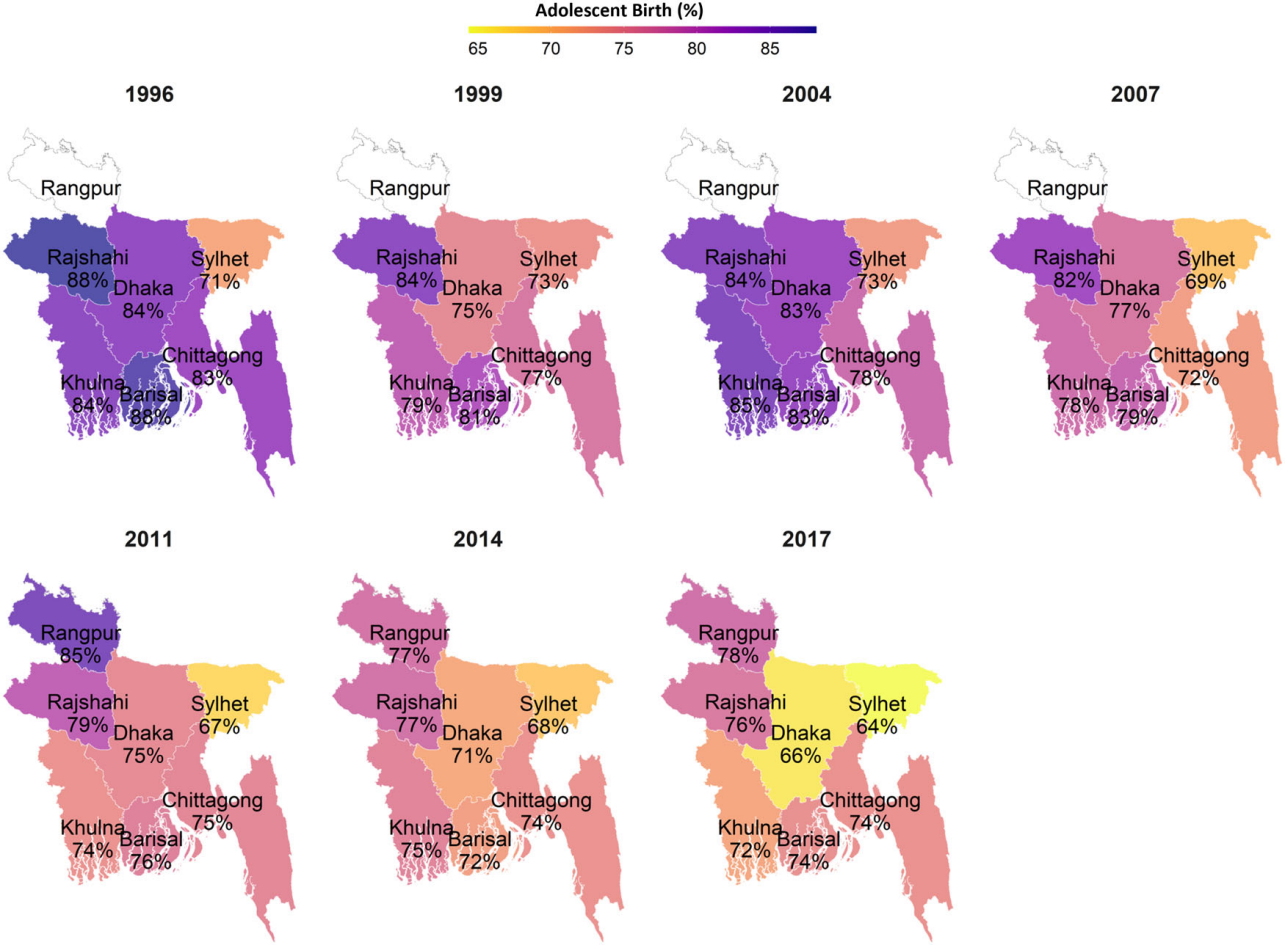
Trends and geographic variability in adolescent birth by survey year, BDHS 1996–2017. Adolescent birth was defined as women giving first birth before 20 years of age among mothers with living children born in the last 5 years. Data were from seven rounds of Bangladesh Demographic and Health Surveys in 1996, 1999, 2004, 2007, 2011, 2014, and 2017. Rangpur Division was formed in 2010. Before that, it belonged to the Rajshahi Division. Thus, no data are available for Rangpur before 2010.

At the national level, the prevalence of first births occurring in women aged 10–19 years old has decreased slowly in Bangladesh from 84% in 1996 to 71% in 2017 (by 13 percentage points (pp); Fig. 1). However, the rate of reduction varied by survey year and age group. Between 1996 and 1999, there was a 6‐pp reduction, following by an increase by 2004 (from 78% to 82%), and then a gradual decrease from 2004 to 2017. The reduction in first births among women aged 10–15 years old was especially large, with the prevalence in 2017 (16%) being less than half of the prevalence in 1996 (35%) in this group. The rate of reduction also varied by division of the country, though no division had a prevalence lower than 64% for any year. The largest reductions occurred in Dhaka (18 pp), Barisal (14 pp), and Rajshahi (12 pp), with smaller reductions in other divisions (ranging from 7 pp in Sylhet to 9 pp in Chittagong; Fig. 2). The prevalence of adolescent birth was lowest in Sylhet for several survey rounds. Rangpur division, for which data were only available for 2011, 2014, and 2017, showed a decrease in 7 pp between those survey rounds.

### Trends in child undernutrition in Bangladesh between 1996 and 2014

**Figure 3:**
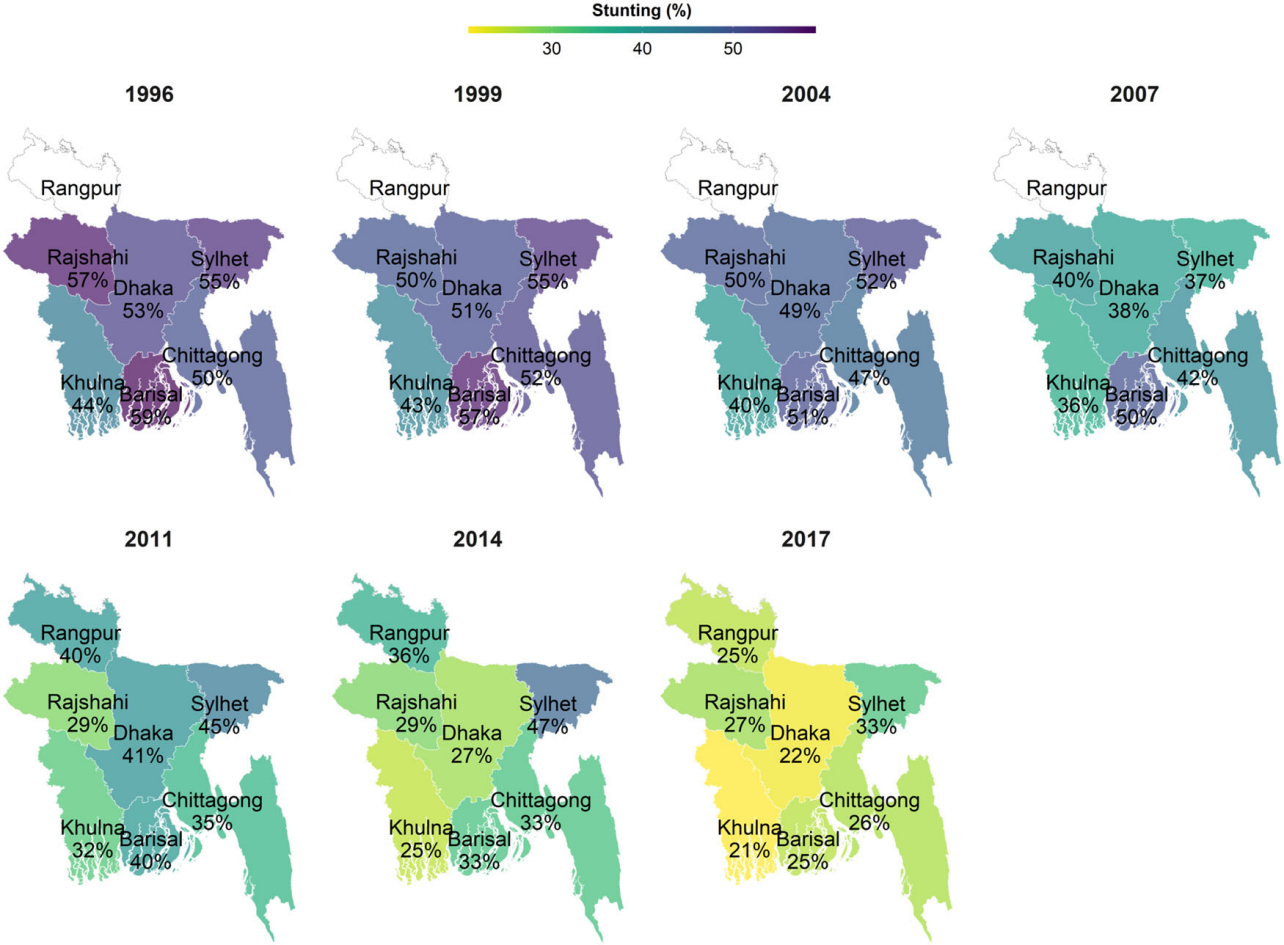
Trends and geographic variabilty in child stunting by survey year, BDHS 1996–2017. This map is based on all children <5 years old (*n* = 44,303). Child stunting was defined as length/height‐for‐age Z‐score <−2. Data were from seven rounds of Bangladesh Demographic and Health Surveys in 1996–1997, 1999, 2004, 2007, 2011, 2014, and 2017. Data are not available for Rangpur in 1996–1997, 1999, 2004, and 2007.

**Figure 4:**
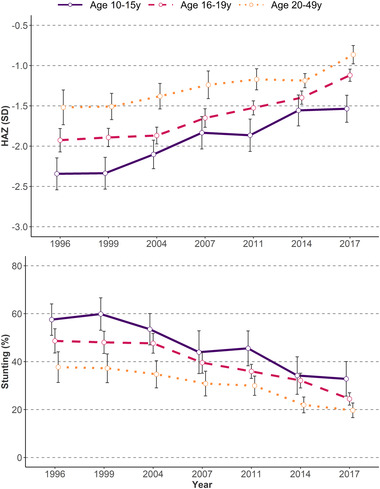
Height‐for‐age Z‐score and stunting by mother's age at first birth and survey year, BDHS 1996–2017. The error bars represent 95% confidence intervals.

Stunting decreased from 59.5% in 1996 to 26.6% in 2017 (by 32.9 pp) at the national level (Table S2, online only), with the highest reduction between 2014 and 2017 (by 9.5 pp). There was heterogeneity across regions (Fig. 3), with Dhaka (38 pp) and Barisal (36 pp) showing the highest reductions, while Sylhet and Khulna showed the lowest reductions (by 28–29 pp). Similar trends were observed for underweight (with a reduction of 32.7 pp from 59.5% in 1996 to 26.6% in 2017) and wasting (with a reduction of 12.9 pp from 21.4% in 1996 to 8.5% in 2017). Over the same time period, child HAZ and WAZ also increased for all age groups (Fig. 4 and Fig. S1, online only).

### Associations between adolescent birth and known determinants of child undernutrition

There were significant associations between maternal age at first birth and various determinants of child nutritional status (Table 1). Adolescent birth was negatively associated with women's nutritional status. Compared with women who first gave birth as adults, women who had their first birth during young adolescence were shorter (−1.0 cm), weighed less (−4.8 kg), had lower BMI (−1.8), and were more likely to be underweight (BMI < 18.5 kg/m^2^, +11.3 pp).

**Table 1:** Association between maternal age at first birth and known determinants of child nutrition, BDHS 1996–2017 (*n* = 12,006)

	First birth during 10–15 years old	First birth during 16–19 years old	First birth during ≥20 years old	10–15 years old versus ≥20 years old*^a^*		16–19 years old versus ≥20 years old*^a^*	
	Mean/%	Mean/%	Mean/%	β	95 % CI	β	95 % CI
Maternal nutritional status							
Height, cm	150.30	151.02	151.37	−0.97***	[−1.32,−0.62]	−0.38**	[−0.63,−0.12]
Weight, kg	44.11	46.44	50.05	−4.75***	[−5.26,−4.25]	−3.42***	[−3.81,−3.03]
BMI, kg/m^2^	19.51	20.33	21.82	−1.82***	[−2.02,−1.62]	−1.40***	[−1.55,−1.24]
BMI < 18.5 kg/m^2^	37.29	31.20	21.49	11.32***	[8.29,14.36]	8.97***	[7.06,10.88]
Living conditions							
SES index	2.69	3.06	3.65	−0.90***	[−0.99,−0.81]	−0.57***	[−0.63,−0.51]
Improved sanitation	46.79	52.20	61.31	−14.90***	[−18.48,−11.32]	−9.49***	[−11.74,−7.25]
Education and bargaining power							
Secondary school education or higher	45.11	63.16	74.37	−24.09***	[−27.11,−21.06]	−11.14***	[−13.19,−9.10]
Decision making^*b*^	39.67	43.61	49.82	−9.84***	[−13.07,−6.60]	−6.25***	[−8.15,−4.35]
Access to health and nutrition services							
4+ ANC visits	16.73	28.90	45.46	−23.99***	[−26.96,−21.02]	−15.74***	[−18.08,−13.39]
Institutional delivery	16.77	29.45	51.94	−27.27***	[−30.37,−24.18]	−20.94***	[−23.27,−18.61]
Skilled birth attendant	19.55	33.96	56.42	−29.16***	[−32.36,−25.96]	−20.91***	[−23.36,−18.47]
Postnatal care	19.45	22.48	34.11	−16.62***	[−20.98,−12.26]	−12.35***	[−14.70,−10.00]
Full immunization	77.82	82.77	87.52	−6.28***	[−9.13,−3.42]	−4.63***	[−6.54,−2.71]
Child vitamin A	66.84	69.86	71.52	−6.45**	[−10.72,−2.17]	−1.94	[−4.35,0.46]
Child IFA	4.34	4.26	7.27	−2.82*	[−4.97,−0.66]	−2.93***	[−4.42,−1.45]
Child deworming	44.23	41.58	44.96	−3.15	[−7.87,1.56]	−3.94**	[−6.76,−1.12]
IYCF practices							
Early initiation of breastfeeding	33.52	42.14	39.98	0.70	[−4.04,5.43]	3.44*	[0.41,6.47]
Adequate diet	24.53	26.04	32.72	−10.14*	[−19.15,−1.13]	−6.57**	[−10.51,−2.63]
Iron‐rich food	60.38	53.11	57.93	2.63	[−6.27,11.53]	−3.72	[−8.35,0.91]

Note: Data were from BDHS 1996–2017.

^*a*^ Ordinary least squares (OLS) regression models were adjusted; for maternal outcomes: adjusted for maternal religion, survey years, and region fixed effects. For child outcomes: adjusted for child age, gender, birth order, maternal religion, survey years, and region fixed effects. All models controlled for the cluster sampling design and sampling weights used in the survey.

^*b*^ Decision making includes women have the ability to decide on spending respondents' earnings, health care, household purchases, and visiting family or relatives.

ANC, antenatal care; BMI, body mass index; IFA, iron and folic acid; IYCF, infant and young child feeding; SES, socioeconomic status.

*** *P* < 0.001; ^**^ *P* < 0.01; ^*^ *P* < 0.05.

Adolescent birth was also negatively associated with living conditions, women's education, and bargaining power. Compared with adult women, women who gave birth during young adolescence were more likely to live in a household with lower SES (−0.9 SD) and poorer sanitation (−14.9 pp); and they had lower education (−24.1 pp for secondary school education) and less household decision‐making power (−9.8 pp).

Adolescent birth was negatively associated with access to services during pregnancy, delivery, and early postpartum. Compared with women who first gave birth as adults, those who gave first birth during young adolescence reported poorer use of antenatal care (−24 pp for 4+ antenatal care visits), less service use during delivery (−27.3 pp for institutional delivery and −29.2 pp for skilled birth attendant) and postnatal care (−16.6 pp), and less access to full immunization (−6.3 pp) and child vitamin A supplementation (−6.5 pp). Finally, adolescent birth was negatively associated with complementary feeding practices (−8.5 pp for adequate diet). Similar associations were found for the comparison of old adolescent versus adult births, with a smaller magnitude of the differences.

### Associations between adolescent birth and childhood undernutrition

Children of women who had their first child during adolescence had lower HAZ and WAZ, as well as higher stunting and underweight prevalence, compared with children of women who had their first child during adulthood. The multivariable regression that adjusted for known determinants of child undernutrition showed that children born to old adolescent mothers (16–19 years old) had lower HAZ (−0.10 SD) and WAZ (−0.06 SD) than children born to adults (20–49 years old) (Table 2). These differences were greater when comparing children born to young adolescents (10–15 years old) with those born to adults (mean difference: −0.29 SD for HAZ and −0.18 for WAZ). Similar findings were seen for stunting (+5.9 pp) and underweight (+6.0 pp) in children born to young adolescent versus adult mothers (Table S3, online only). No associations were observed for adolescent birth and WHZ or wasting.

**Table 2:** Association between mother's age at first birth and child height‐for‐age Z‐score (HAZ), weight‐for‐age Z‐score (WAZ), and weight‐for‐height Z‐score (WHZ), BDHS 1996–2017 (*n* = 12,006)

	HAZ^*a*^		WAZ^*a*^		WHZ^*a*^	
	β	[95% CI]	β	[95% CI]	β	[95% CI]
Adolescent birth^*b*^						
First birth during 10–15 years old	−0.29***	[−0.38,−0.20]	−0.18***	[−0.26,−0.10]	−0.03	[−0.12,0.06]
First birth during 16–19 years old	−0.10***	[−0.16,−0.04]	−0.06*	[−0.11,−0.00]	0.01	[−0.05,0.07]
Maternal nutritional status						
Height, cm	0.05***	[0.04,0.05]	0.03***	[0.02,0.03]	−0.00	[−0.01,0.00]
Weight, kg	0.02***	[0.02,0.02]	0.03***	[0.03,0.03]	0.03***	[0.02,0.03]
Living conditions						
SES index	0.09***	[0.06,0.11]	0.06***	[0.04,0.09]	0.02*	[0.00,0.05]
Improved sanitation	−0.02	[−0.08,0.04]	0.02	[−0.03,0.07]	0.04	[−0.02,0.09]
Education and bargaining power						
Secondary school education or higher	0.21***	[0.15,0.27]	0.17***	[0.12,0.23]	0.07*	[0.00,0.13]
Decision making*^c^*	0.12*	[0.01,0.23]	0.09*	[0.01,0.17]	0.02	[−0.07,0.11]
Access to health and nutrition services						
4+ ANC visits	0.09**	[0.03,0.16]	0.12***	[0.06,0.18]	0.08*	[0.01,0.15]
Skilled birth attendant	0.28***	[0.21,0.35]	0.27***	[0.19,0.34]	0.13**	[0.04,0.22]
Postnatal care	−0.10*	[−0.19,−0.02]	−0.12**	[−0.21,−0.04]	−0.08	[−0.18,0.01]
Full immunization	0.18***	[0.08,0.27]	0.16***	[0.09,0.24]	0.07	[−0.02,0.15]
Child vitamin A	−0.06	[−0.13,0.02]	−0.04	[−0.10,0.03]	−0.03	[−0.10,0.05]
Child IFA	−0.04	[−0.19,0.12]	−0.02	[−0.16,0.12]	−0.02	[−0.18,0.14]
Child deworming	−0.14**	[−0.23,−0.05]	−0.12**	[−0.19,−0.04]	−0.09*	[−0.17,−0.01]
IYCF practices						
Early initiation of breastfeeding	0.06	[−0.01,0.13]	0.12***	[0.05,0.19]	0.08*	[0.01,0.15]
Adequate diet	0.04	[−0.09,0.17]	0.04	[−0.09,0.17]	0.04	[−0.10,0.19]
Iron‐rich food	0.05	[−0.08,0.18]	0.17**	[0.05,0.29]	0.15*	[0.02,0.29]

Note: Data were from BDHS 1996–2017.

^*a*^ Regression models were adjusted for child age, gender, maternal religion, region fixed effects, and the cluster sampling design and sampling weights used in the survey.

^*b*^ The association between maternal age at first birth and child HAZ, WAZ, and WHZ was also adjusted for all the other indicators in the table (maternal nutritional status, education, living conditions, access to health services, and complementary feeding).

^*c*^ Decision making includes women have the ability to decide on spending respondents' earnings, health care, household purchases, and visiting family or relatives.

ANC, antenatal care; BMI, body mass index; IFA, iron and folic acid; IYCF, infant and young child feeding; SES, socioeconomic status.

*** *P* < 0.001; ^**^ *P* < 0.01; ^*^ *P* < 0.05.

## Discussion

Using data from multiple nationally representative health surveys, we aimed to examine trends in adolescent birth and child undernutrition in Bangladesh in the last two decades and to quantify the magnitude of the association between adolescent birth and child undernutrition after accounting for potential factors that may underlie this relationship. We found that the prevalence of adolescent birth decreased modestly between 1996 and 2017 (by 13 pp) but remained high (at 71.4%). Child undernutrition improved over time (by 23 pp for stunting and 36 pp for underweight), but poor growth was more prevalent among children born to adolescent mothers. The risk of being stunted and underweight increased 5.9 and 6.0 pp, while HAZ and WAZ reduced by 0.29 and 0.18 SD, respectively, even after controlling for known determinants of undernutrition, such as maternal nutritional status, education, living conditions, access to health services, and complementary feeding practices.

The multiple linkages between adolescent birth and child undernutrition observed in this Bangladesh study align well with a previous study conducted in India.^9^ Both studies found that compared with women who first gave birth as adults, those who first gave birth as adolescents were shorter and thinner, lived in poorer households with poorer sanitation, and had lower access to health services, poorer complementary feeding practices, and lower education. Because first births occur earlier in Bangladesh than in India (17.3 versus 18.3 years old), a new contribution of this analyses was being able to compare the magnitude of the association between child growth and giving birth in young (10–15 years old) and old (16–19 years old) adolescence. We found that poor child growth was much more pronounced in women who have children as young adolescents compared with women who have children as old adolescents. Estimates from DHS data in 42 countries showed around 2.5 million births occur to girls aged 12–15 years old in low‐resource countries each year, and the health risks (both morbidity and mortality) associated with adolescent birth are concentrated among the youngest girls.^31^

Adolescence is a period of rapid physiological, sexual, neurological, and behavioral changes; the growth spurt in adolescence requires increased energy and nutrients.^32^ Pregnancy during adolescence compounds the higher nutritional needs of adolescent girls who have not completed their own growth and development, increasing their risk of being shorter, thinner, and/or having depleted stores of energy and micronutrients. A previous study in Bangladesh showed that pregnancy and lactation during adolescence halts linear growth and results in weight loss and depletion of fat and lean body mass,^10^ while other studies showed that pregnant teens continue to grow during gestation.^33^, ^34^ Becoming pregnant during this rapid phase of growth leads to the competition for nutrients between the mother and fetus,^32^ a battle that the fetus invariably loses.^35^ In situations where maternal nutritional status is depleted, the partitioning would favor the mother over the fetus, leading to several adverse pregnancy outcomes, including low birth weight, preterm delivery, and severe neonatal conditions, which may carry long‐term consequences.^15^

Beyond increased nutritional needs, adolescent mothers face several other barriers to achieving optimal pregnancy outcomes. Consistent with previous studies,^9^, ^36^ our findings show that adolescent mothers had lower access to and utilization of maternal health and nutrition services across the continuum of care, and these factors serve as an important link between adolescent pregnancy and child nutrition. In addition to the challenges of psychological transition from childhood to adulthood and limited reproductive knowledge,^37^ previous studies highlighted low autonomy, SES, and decision‐making power as important sociocultural barriers to utilization of health services for young mothers in Bangladesh.^36^, ^38^ Our finding that adolescent birth was independently associated with child stunting and underweight even after adjusting for a set of selected determinants suggests a mechanism beyond these determinants. One explanation may be that adolescent mothers have short pregnancies; findings from a multicountry study showed that adolescent mothers are more likely to deliver preterm and low birth weight babies compared with adult mothers.^15^ Behavioral mechanisms are also likely at play. Adolescents are still undergoing their own complex life transition, and inadequacy of resources for their own mental and physical care during pregnancy and their infant's care during the first few years of life could result in poor child developmental outcomes.^39^ These complex mechanisms highlight the importance of a life‐course framework to prevent early pregnancy and negative pregnancy‐related outcomes that includes upstream programs (a package of promotive and preventive interventions for school‐age girls and boys even before reproductive maturity), midstream programs (a package of promotive, preventive, and curative interventions in the prepregnancy period), and downstream programs (during antenatal, childbirth, and postnatal periods).^40^

A major cause of adolescent pregnancies in Bangladesh is child marriage—that is, marriage before the legal age of 18 years old, which is highly prevalent in the country (∼70%).^6^ Despite years of social movements to prevent child marriage in Bangladesh, the problem persists because of strong social norms in relation to religion, social pressure, perceived gendered roles of girls, maintenance of family honor and chastity of girls, pressure of a dowry, and concerns for safety and security.^41^, ^42^, ^43^ Once married, couples are encouraged to have children because of perceptions that delaying the first birth after marriage could result in being a “childless woman,” rumors of infertility, shame on the family, and loss of the husband because the husband's family wishing to seek another wife for their son.^42^ Child marriage, early childbearing, and poor nutrition are interlinked and share drivers, including household poverty, women's disempowerment, and deeply embedded social norms, requiring convergence of existing effective and specific interventions to tackle the issues.

Drawing lessons learned from global evidence,^44^, ^45^ Bangladesh programs for adolescent health have used several strategies, including empowering girls with information, skills and support networks; mobilizing families and communities; increasing access to quality education; incentivizing families through economic support; and strengthening the policy framework around early marriage. Schools in particular have been leveraged as delivery platforms for adolescent‐focused interventions. School feeding programs in Bangladesh tend to be limited to primary schools. While primary school enrollment is almost universal in Bangladesh, secondary school dropout rates are high, especially for girls. One option for reducing adolescent pregnancy is considering a secondary school meals program combined with conditional cash transfers. This program would provide nutritious school meals to all secondary school girls and boys, and target cash transfers only to adolescent girls from poor families, conditional on not getting married. Girls in higher grades can receive a larger amount of cash to prevent them from dropping out. Prominent examples of programs aiming to reduce adolescent marriage include the Female Secondary School Stipend Program (1992–1996), which introduced uniform stipends and tuition subsidy programs to girls attending secondary schools in rural areas;^46^ the KAISHAR program (2002–2008), which disseminated information on adolescent reproductive and sexual health to communities, trained health providers to provide services to adolescents, and equipped adolescent information centers;^47^ and the BALIKA program (2014–2015) which provided either education support, life skills training, or livelihoods training to adolescents.^48^

For girls who are already married, the focus should be on delaying pregnancy. To this end, massive social campaigns, similar to what Bangladesh has very successfully done for child immunization, could be effective for encouraging family planning to delay pregnancy for adolescent girls. While many interventions show modest impacts on delaying age at marriage, few programs have been able to enable adolescent mothers to continue their education or increase their access to reproductive health services,^49^ and none, to our knowledge, have documented impacts on child nutrition. Programs to reduce early childbearing often work through reproductive health services, while programs to reduce undernutrition operate through maternal and child health program. Effective convergence of interventions is needed in efforts to reduce early marriage and birth; these efforts will simultaneously improve adolescent maternal and child nutrition.

In order to achieve the SDG target to end child marriage by 2030, the pace of early marriage reduction would need to be eight times faster than what was observed in the last decade.^50^ The COVID‐19 pandemic poses additional challenges that may increase child marriage, including school closures, constrained access to education and health services, increasing poverty and food insecurity, and exposure to violence.^51^ It is estimated that an additional 2.5 million girls will be at risk of early marriage over the next 5 years, and adolescent pregnancies are expected to rise by up to 1 million in 2020 globally, with Bangladesh contributing a large share.^51^ Disruption in sexual and reproductive health care services is projected to affect 48 million additional women with an unmet need for modern contraceptives and contribute to 15 million additional unwanted pregnancies annually.^52^ Innovative and strong multisectoral strategies are needed to address the multiple consequences of COVID‐19 on early marriage and childbearing, and to improve the wellbeing and livelihoods of adolescents.

Our study has multiple strengths, including large sample size from multiple nationally representative surveys, advanced statistical methods, and the disaggregation of results for younger and older adolescent mothers. By using multiple rounds of DHS data between 1996 and 2017, we present trends over the last two decades and show robust associations between adolescent birth and child undernutrition as well as determinants of child undernutrition across survey rounds. Our study is one of very few studies that have disaggregated outcomes for adolescent mothers by age, which provides in‐depth insights into the detrimental effects of very early childbearing (by 15 years of age). We acknowledge some limitations of the study, including the use of repeated cross‐sectional data, which prevents us from drawing causal inferences. We are also not able to examine how social norms have changed over time and the direct or indirect roles of these unobserved factors.

In conclusion, adolescent birth is still the norm in Bangladesh, and it has implications for the health, nutritional, and SES of mothers as well as children. These findings call for effective convergence of interventions across multiple sectors at the right time in life to address these persistent problems. Targeting meaningful reductions in the number of women marrying and having children as adolescents will contribute to the attainment of several SDGs, including those addressing poverty, hunger, good health and wellbeing, inclusive and quality education, and gender equality.

## Author contributions

P.H.N. conceived the idea for the manuscript and led the overall synthesis of the manuscript, conducted the statistical analysis, and wrote significant sections of the manuscript. S.S. helped conceive the idea for the manuscript, conducted literature review, reviewed the statistical analyses, and wrote significant sections of the manuscript. L.Q.K. conducted the statistical analysis and prepared tables and figures for the manuscript. P.P. conducted literature review and edited the manuscript. A.A., S.F.R., and K.A. supported data interpretation and reviewed and edited the manuscript. P.M. reviewed the statistical analyses, supported data interpretation, and reviewed and edited the manuscript. All authors read and approved the final submitted manuscript.

## Competing interests

The authors declare no competing interests.

## Supporting information




**Table S1**. Descriptive characteristics of participants in seven BDHS 1996–2017.
T**able S2**. Trends in adolescent birth and child undernutrition in seven BDHS 1996–2017.
**Table S3**. Association between mother's age at first birth and child stunting, underweight, and wasting by survey year, BDHS 1996–2017 (*n* = 12,006).
**Figure S1**. Weight‐for‐age Z‐score, weight‐for‐height Z‐score, underweight, and wasting by mother's age at first birth and survey year, BDHS 1996–2017 (*n* = 12,006).
