# Supplementary material for: Adolescent birth and child undernutrition: an analysis of demographic and health surveys in Bangladesh, 1996–2017
Source: Ann N Y Acad Sci. 2021 May 14;1500(1):69–81. doi: 10.1111/nyas.14608 (PMC8518722; doi:10.1111/nyas.14608)
Supplement: Supplementary file 1 — Table S1. Descriptive characteristics of participants in seven BDHS 1996–2017. Table S2. Trends in adolescent birth and child undernutrition in seven BDHS 1996–2017. Table S3. Association between mother's age at first birth and child stunting, underweight, and wasting by survey year, BDHS 1996–2017 (n = 12,006). Figure S1. Weight‐for‐age Z‐score, weight‐for‐height Z‐score, underweight, and wasting by mother's age at first birth and survey year, BDHS 1996–2017 (n = 12,006). [file NYAS-1500-69-s001.pdf]

Supplemental Table 1. Descriptive characteristics of participants in 7 Bangladesh Demographic and Health Surveys, 1996- 2017

	1996	1999	2004	2007	2011	2014	2017	Pooled data
**Sample**								
Number of clusters	313	341	361	361	600	600	672	3248
Number of households	8,682	9,854	10,500	10,400	17,141	17,300	19,457	93,334
Number of HH with living children <5y	4,289	4,775	4,941	4,582	6,829	6,429	7,057	31,845
Number of all children <5y having anthropometric data	4,898	5,431	6,002	5,349	7,742	7,031	7,850	44,303
Number of primiparous women with children <5y having anthropometric data	995	1,154	1,359	1,376	2,250	2,340	2,532	12,006
**Household**								
Place of residence (rural)	90.7	83.6	80.3	79.0	77.9	74.8	73.5	79.5
Religion as Islam, %	91.8	89.7	92.1	91.4	91.4	91.4	91.7	91.1
**Mother**								
Age, y	25.8	25.8	25.6	25.6	25.5	25.5	25.7	25.6
Age at marriage, y	14.2	15.0	14.9	15.4	15.8	16.3	16.4	17.7
Age at first birth, y	17.0	17.6	17.3	17.8	17.9	18.2	18.4	15.4
In work force, %	33.9	17.5	17.6	26.8	9.1	26.2	40.6	21.4
**Child**								
Age, month	28.8	28.4	29.2	29.5	30.3	29.2	28.5	26.3
Female child, %	50.4	49.3	49.2	50.2	49.1	48.0	47.8	48.7

Supplemental Table 2. Trend in adolescent birth and child undernutrition in 7 Bangladesh Demographic and Health Surveys, 1996- 2017

	1996	1999	2004	2007	2011	2014	2017	Overall test for trend
**Adolescent birth**								
First birth at 10-15y, %	35.3	26.3	29.5	23.9	21.6	18.7	15.5	<0.001
First birth at 16-19y, %	48.6	51.7	52.0	52.7	54.2	54.5	56.0	<0.001
First birth at 10-19 y, %	83.9	78.1	81.5	76.6	75.9	73.2	71.4	<0.001
First birth at 20-24y, %	13.8	18.7	15.9	20.1	19.9	22.2	23.9	<0.001
First birth at 25-49y, %	2.3	3.2	2.6	3.3	4.2	4.6	4.7	<0.001
First birth at 20-49y, %	16.1	21.9	18.5	23.4	24.1	26.8	28.6	<0.001
**Child undernutrition**								
All child <5y								
HAZ, mean ± SD	-2.29 ± 1.58	-1.98 ± 1.44	-1.95 ± 1.37	-1.76 ± 1.38	-1.63 ± 1.45	-1.53 ± 1.32	-1.17 ± 1.39	<0.001
WAZ, mean ± SD	-2.05 ± 1.25	-1.76 ± 1.13	-1.78 ± 1.13	-1.74 ± 1.13	-1.58 ± 1.18	-1.48 ± 1.12	-1.03 ± 1.16	<0.001
WHZ, mean ± SD	-1.03 ± 1.39	-0.87 ± 1.09	-0.93 ± 1.16	-1.04 ± 1.12	-0.93 ± 1.26	-0.89 ± 1.17	-0.53 ± 1.19	<0.001
Stunting, %	59.5	50.3	49.8	44.0	40.3	36.1	26.6	<0.001
Underweight, %	52.3	41.2	42.3	41.6	35.8	32.5	19.6	<0.001
Wasting, %	21.4	12.6	14.8	17.6	15.8	14.6	8.5	<0.001

Adolescent birth was defined as women giving first birth before 20 years of age among mothers with living children born in the last 5 years. Data were from 7 rounds of Bangladesh's Demographic and Health Survey (DHS) 1996, 1999, 2004, 2007, 2011, 2014 and 2017. HAZ: height-for-age z-score; WAZ: weight-for-age z-score; WHZ: weight-for-height z-score.

Supplemental Figure 1. Weight-for-age z-score, weight-for-height z-score, underweight and wasting by mother's age at first birth and survey year, Bangladesh Demographic Health Survey 1996–2017 (N = 12,006)

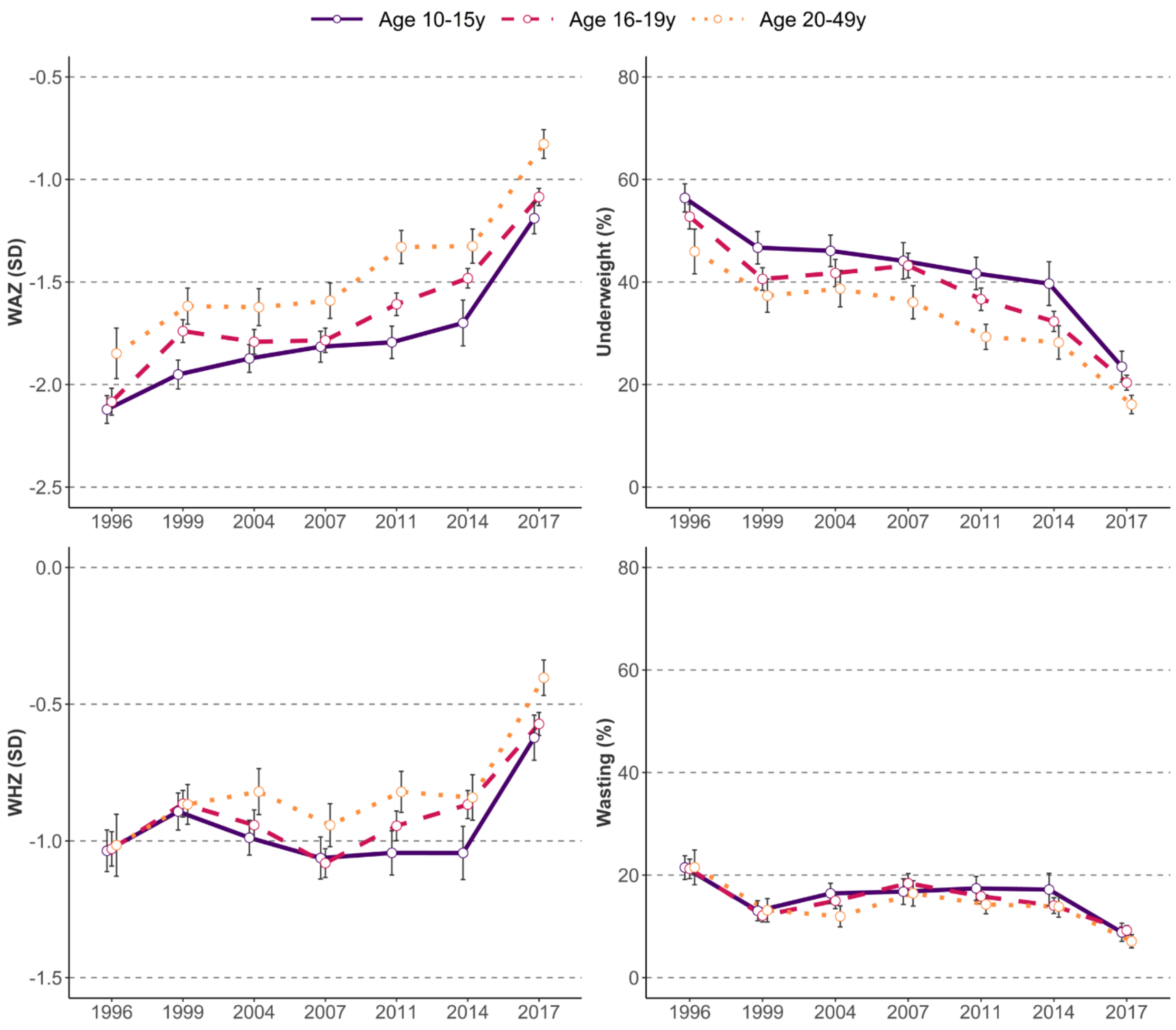

The error bars represent 95% confidence intervals.

Supplemental Table 3. Association between mother's age at first birth and child stunting, underweight, and wasting by survey year, Bangladesh Demographic Health Survey 1996–2017 (N = 12,006)

	Stunting		Underweight		Wasting	
	β	*[95% CI]*	*β*	*[95% CI]*	*β*	*[95% CI]*
**Adolescence birth**						
First birth during 10-15 y	5.85***	[2.55,9.14]	6.01***	[2.80,9.23]	1.12	[-1.34,3.58]
First birth during 16-19 y	2.02	[-0.09,4.13]	0.58	[-1.48,2.64]	-0.07	[-1.62,1.47]
**Maternal nutritional status**						
Height, cm	-1.52***	[-1.70,-1.33]	-0.82***	[-1.00,-0.64]	0.04	[-0.10,0.18]
Weight, kg	-0.51***	[-0.64,-0.39]	-0.81***	[-0.93,-0.69]	-0.40***	[-0.48,-0.31]
**Living condition**						
SES index	-2.25***	[-3.13,-1.37]	-1.72***	[-2.53,-0.90]	-0.21	[-0.84,0.42]
Improved sanitation	-1.84	[-3.95,0.28]	0.09	[-1.92,2.10]	-0.07	[-1.57,1.43]
**Education and bargaining power**						
Education ≥ secondary	-7.20***	[-9.51,-4.88]	-6.48***	[-8.72,-4.24]	-3.44***	[-5.22,-1.65]
Decisions making[1]	-1.78	[-5.65,2.08]	-0.86	[-4.17,2.44]	-0.85	[-3.48,1.79]
**Access to health and nutrition services**						
4+ ANC	-3.04*	[-5.37,-0.71]	-3.21**	[-5.47,-0.96]	0.97	[-0.82,2.77]
Skilled birth attendant	-8.87***	[-11.44,-6.30]	-5.73***	[-8.30,-3.15]	-3.38**	[-5.45,-1.31]
Postnatal care	3.82*	[0.78,6.86]	3.48*	[0.71,6.25]	3.51**	[1.19,5.83]
Full immunization	-4.96**	[-8.09,-1.84]	-4.16**	[-7.20,-1.12]	-0.27	[-2.77,2.24]
Child Vitamin A	3.28**	[0.80,5.75]	3.02*	[0.63,5.41]	1.63	[-0.33,3.59]
Child IFA	0.32	[-5.64,6.27]	3.82	[-2.22,9.86]	0.87	[-3.92,5.65]
Child deworming	3.12	[-0.17,6.41]	3.25*	[0.09,6.41]	0.56	[-1.66,2.78]
**IYCF practices**						
Early initiation of breastfeeding	-1.33	[-3.98,1.32]	-3.13*	[-5.77,-0.49]	-0.94	[-2.91,1.03]
Adequate diet	-1.74	[-6.31,2.83]	-5.34*	[-9.92,-0.75]	0.49	[-2.82,3.80]
Iron rich food	-0.27	[-4.94,4.39]	-2.29	[-7.22,2.64]	-1.95	[-5.69,1.78]

Regression models are adjusted for child age, gender, maternal religion, region fixed effects, the cluster sampling design and sampling weights used in the survey, ANC: antenatal care, IFA: iron and folic acid, SES: Socio-economic status. [1]Decision making includes: Women have the ability to decide on: spending respondent's earnings, health care, household purchases, visiting family or relatives. Data were from Bangladesh's Demographic and Health Survey (DHS) 1996- 2017. *** p<0.001, ** p<0.01, * p<0.05.
